# Tempol Protects Against Acetaminophen Induced Acute Hepatotoxicity by Inhibiting Oxidative Stress and Apoptosis

**DOI:** 10.3389/fphys.2019.00660

**Published:** 2019-05-31

**Authors:** Zheng Ge, Chenyu Wang, Junjie Zhang, Xiwang Li, Junhong Hu

**Affiliations:** Department of General Surgery, Huaihe Hospital of Henan University, Kaifeng, China

**Keywords:** acetaminophen, acute hepatotoxicity, tempol, oxidative stress, apoptosis

## Abstract

Acetaminophen (APAP)-induced acute hepatotoxicity is the leading cause of drug-induced acute liver failure. The aim of this study was to evaluate the effects of 4-hydroxy-2,2,6,6-tetramethylpiperidine-N-oxyl (tempol) on the protection of APAP-induced hepatotoxicity in mice. Mice were pretreated with a single dose of tempol (20 mg/kg per day) orally for 7 days. On the seventh day, mice were injected with a single dose of APAP (300 mg/kg) to induce acute hepatotoxicity. Our results showed that tempol treatment markedly improved liver functions with alleviations of histopathological damage induced by APAP. Tempol treatment upregulated levels of antioxidant proteins, including superoxide dismutase, catalase, and glutathione. Also, phosphorylation of phosphoinositide 3-kinase (PI3K) and protein kinase B (Akt) and protein expression of nuclear factor erythroid 2-related factor (Nrf 2) and heme oxygense-1 (HO-1) were all increased by tempol, which indicated tempol protected against APAP-induced hepatotoxicity *via* the PI3K/Akt/Nrf2 pathway. Moreover, tempol treatment decreased pro-apoptotic protein expressions (cleaved caspase-3 and Bax) and increased anti-apoptotic Bcl-2 in liver, as well as reducing apoptotic cells of TUNEL staining, which suggested apoptotic effects of tempol treatment. Overall, we found that tempol normalizes liver function in APAP-induced acute hepatotoxicity mice *via* activating PI3K/Akt/Nrf2 pathway, thus enhancing antioxidant response and inhibiting hepatic apoptosis.

## Introduction

Acetaminophen (APAP) is a widely used drug for its antipyretic and analgesic properties. It was reported that APAP-induced acute hepatotoxicity is the most common cause of drug-induced acute liver injury and liver failure in many countries (Canbay et al., 2009; Blieden et al., 2014). Previous studies used 300 mg/kg APAP to induce acute liver injury in mice to explore the underlying pathogenesis (Huang et al., 2017; Liao et al., 2017; Cai et al., 2018). However, the detailed mechanisms of APAP-induced acute liver injury are still not fully clear. It was hypothesized that many pathological events are involved in the process, including oxidative stress, endoplasmic reticulum stress, apoptosis, autophagy, inflammation, and liver regeneration. Great efforts should be made to further understand the exact mechanism of APAP-induced liver toxic effects.

Tempol (4hydroxy-2,2,6,6-tetramethylpiperidine-N-oxyl) is a water-soluble, heterocyclic compound and concerned as a small membrane permeable superoxide dismutase (SOD) mimetic (Wilcox, 2010). It was regarded as a beneficial antioxidant and radical scavenger (Pinar et al., 2017). Tempol has been proven to own various protective effects among diseases, including hypertension (Wilcox, 2010), cardiac ischemia/reperfusion injury (Gelvan et al., 1991), gastrointestinal damage (Deguchi et al., 2011), and inflammation (Wilcox and Pearlman, 2008). Nonetheless, few studies have evaluated the effects of tempol on the protection of APAP-induced acute liver injury.

Nuclear factor erythroid 2-related factor (Nrf 2) is reported to be activated by redox status changes of the kelch-like ECH associated protein 1 (Keap1) (Wang et al., 2015). Nrf 2 activation leads to regulate antioxidant defense genes and antioxidant enzymes, such as heme oxygense-1 (HO-1) and NADPH:quinone oxidoreductase 1 (NQO-1) (Goldring et al., 2004; Zhang et al., 2010). These antioxidant responses which are activated by Nrf 2 act as cell defense system to protect cell injury. Previous studies suggested that Nrf 2 was vital in the protection against drug-induced liver hepatotoxicity (Gum and Cho, 2013; Jiang et al., 2016). However, whether Nrf 2 is involved in the protection of tempol against APAP-induced acute hepatotoxicity is not fully understood.

In the present study, we used a mouse model of APAP induced acute hepatotoxicity and investigated whether tempol treatment could prevent APAP-related acute hepatic dysfunction and studied underlying signaling mechanisms.

## Materials and Methods

### Experimental Animals and Protocols

Male C57BL/6 mice (8–10 weeks old) were obtained from the Animal Center Laboratory of Henan Province. Mice were housed under climate-controlled conditions with a 12-h light/dark cycle and provided with standard food and water. All experimental procedures were performed in accordance with international guidelines for the care and use of laboratory animals and approved by the Animal Ethics Committee of the Henan University School of Medicine, Kaifeng, China.

The mice were randomly divided into three groups of 10 animals each: (1) normal control group (Control), (2) APAP-treated group (APAP, 300 mg/kg), and (3) APAP/tempol-treated group (APAP + Tempol, 300 mg/20 mg/kg). Acute hepatotoxicity in mice was induced by injection with acetaminophen (APAP, Sigma, St. Louis, MO, USA). Mice were pretreated with a single dose of tempol (20 mg/kg per day) orally for 7 days. On the seventh day, mice were injected with a single dose of APAP (300 mg/kg) after pre-administered of tempol for 1 h. After 24 h, all mice were prepared for experiments.

### Measurement of Serum ALT and AST

Animals were anesthetized by 3% isoflurane. Blood of mice was collected from the inferior vena cava and centrifuged at 3,000 rpm, 4°C for 15 min, and serum was taken from the supernatant and stored at −80°C until analysis. Serum ALT and AST were measured by an automatic biochemical analyzer (Roche, Germany) according to the manufactures’ protocols.

### Determination of H_2_O_2_, SOD, CAT, MDA, and GSH

Mice were killed by cervical dislocation after collecting blood and the livers were removed, immediately frozen in liquid nitrogen, and stored at −80°C. Then frozen liver tissues were homogenized and centrifuged at 12,000 *g*, 4°C for 8 min. The supernatants were used for measurements. The concentrations of hydrogen peroxide (H_2_O_2_) were measured by using standard assays according to manufacture instructions (Beyotime Biotechnology, China) (Li et al., 2016). Briefly, concentration of H_2_O_2_ of the supernatants was measured by incubation of supernatants with H_2_O_2_ test solutions for 30 min at room temperature and detection of the absorbance at 560 nm. Measurement of catalase activity (CAT) was accorded to the manufacturer’s instructions of catalase analysis kit (Beyotime Biotechnology, China). In this kit, metabolized H_2_O_2_ is coupled to a chromogenic substrate and treated with peroxidase to generate a red product that is quantitated in a spectrometer at a wavelength of 520 nm. One unit of catalase activity was defined as the amount of H_2_O_2_ metabolized per minute at room temperature. SOD activity levels were quantitated as a total superoxide dismutase by a kit (Beyotime Biotechnology, China) based on the chromogenic product of the WST-8. In this kit, water-soluble formazan dye is generated by WST-8 in the presence of superoxide anion driven by xanthine oxidase, detectable by a colorimetric assay, and quantitated in a spectrometer at 450 nm. The concentrations of malondialdehyde (MDA) were measured by using the Lipid Peroxidation MDA assay kit according to manufacture instructions s (Beyotime Biotechnology, China) (Gong and Li, 2011). In this kit, MDA reacts with thiobarbituric acid in a higher temperature and acidic environment to form a red MDA-TBA adduct, then quantitated in a spectrometer at 532 nm. The concentrations of glutathione (GSH) were measured by using standard assays according to manufacture instructions s (Beyotime Biotechnology, China). The method was based on an enzymatic recycling assay described (Anderson, 1985). Briefly, GSH of the liver tissue supernatant can react with the chromogenic substrate DTNB to produce yellow product, the amount of total glutathione determines the amount of yellow TNB formed. Thus, the amount of total glutathione can be calculated at A_412_ by multiscan spectrum.

### Histological Study

To evaluate changes of the liver tissues including central vein congestion, inflammatory cell infiltration, hepatocyte necrosis, and cell apoptosis, the fresh liver tissues were collected and fixed in 4% paraformaldehyde solution for over 24 h. Then liver tissues were dehydrated and paraffin-embedded and sectioned in 5 μm thickness on a Leica Rotary Microtome. Liver sections were stained with hematoxylin-eosin (H&E) for histological assessment using light microscope. Histological damages were graded by an experimenter blinded to treatment group (Liang et al., 2014). TUNEL staining was performed using a cell death detection commercial kit (Thermo Fisher Scientific, MA, USA) in accordance with the manufacturer’s instructions (Henkel et al., 2018). The nuclei were lightly counterstained with hematoxylin. Apoptotic cells with nuclei staining brown were recognized as TUNEL-positive cells excluding necrosis. Numbers of TUNEL-positive tubular cells were quantified by counting 10 randomly chosen, non-overlapping fields per slide. The analysis was done by an experimenter blinded to treatment group. The results were analyzed using Image-Pro plus, version 6.0 (Media Cybernetics). All morphometric analyses were performed in a blinded manner.

### Immunoblotting Analysis

Western-blot analysis was carried out according to our previous report (Ge et al., 2018). Briefly, mice were killed by cervical dislocation after collecting blood and the livers were removed, immediately frozen in liquid nitrogen, and stored at −80°C. Frozen liver tissue samples were homogenized using RIPA buffer containing cocktail proteinase. The homogenate was centrifuged at 12,000 *g* for 8 min and supernatants were collected. Protein concentrations were measured using the BCA protein assay kit (Solarbio, Beijing, China). Equivalent amounts of protein were separated by SDS-polyacrylamide gel electrophoresis and transferred onto polyvinylidene difluoride membranes and were blocked with 5% non-fat dry milk. Membranes were incubated with specific primary antibodies (Abcam, Cambridge, UK): Nrf 2, HO-1, β-actin, Akt, p-Akt (Ser 473), PI3K, p-PI3K (Tyr 458), Bcl-2, Bax, pro-caspase-3, cleaved caspase-3, and Histone H3 followed by incubation with HRP-conjugated anti-mouse/rabbit IgG secondary antibodies (Cell Signaling Technology, Beverly, MA, USA). Bands were visualized by enhanced chemiluminescent substrates (ECL, Thermo Scientific) and analyzed using Image J software (NIH, USA).

### Statistical Analysis

All data are expressed as the mean ± SEM. The significance between the different groups was analyzed by one-way ANOVA followed by a Bonferroni multiple comparison test and considered to be significant at *p* < 0.05. All statistical calculations were made using GraphPad Prism Software 6.0.

## Results

### Tempol Administration Ameliorates Hepatic Dysfunction in Acetaminophen-Induced Acute Hepatotoxicity

**Figure 1:**
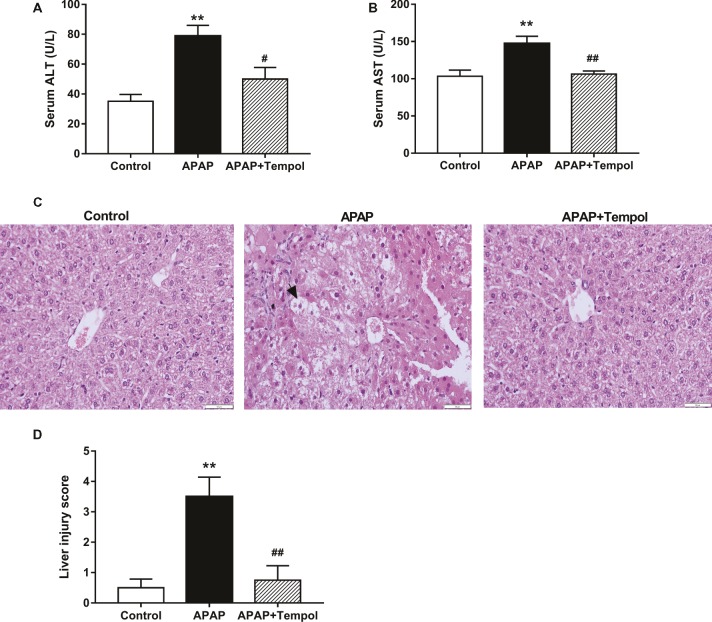
Tempol administration ameliorates hepatocellular damage hepatocellular damage in acetaminophen-induced acute hepatotoxicity. **(A)** Levels of ALT in serum from the control mice (Control), vehicle-treated hepatotoxicity mice (APAP), and tempol-treated hepatotoxicity mice (APAP + tempol). **(B)** Levels of AST in serum from three groups. **(C)** H&E staining of hepatic tissues from three groups. Black arrows indicate hepatocyte necrosis. Scale bar = 50 μm. **(D)** Quantification of acute hepatic injury based on a semi-quantitative morphological scoring system from 0 to 5. Data are mean ± SEM. ^#^*p* < 0.05 vs. APAP, **p < 0.01 vs. control, ^##^*p* < 0.01 vs. APAP, *n* = 6.

We found that tempol treatment significantly improved pathological changes in hepatic function parameters and liver histological changes induced by APAP. Levels of serum glutamic pyruvic transaminase (ALT) and glutamic oxalacetic transaminase (AST) were increased in APAP group compared with control group (Figure 1, ALT, 77.1 ± 14.4 versus 35.1 ± 9.2 U/L; AST, 147.3 ± 19.6 versus 102.2 ± 15.0 U/L; *p* < 0.05). However, tempol-treated APAP group improved the hepatic function parameters compared with vehicle-treated APAP group (Figure 1, ALT, 49.5 ± 15.6 versus 77.1 ± 14.4 U/L; AST, 106.3 ± 8.1 versus 147.3 ± 19.6 U/L; *p* < 0.05). Hematoxylin and eosin stained sections are shown in Figure 1. Histology of liver tissues from the control mice showed a normal liver lobular architecture with cords of hepatocytes radiating from the central vein. By contrast, the APAP treatment exhibited severe histopathological changes such as swollen centrilobular hepatocytes with highly vacuolated cytoplasm, large areas of extensive cell necrosis with loss of hepatocyte architecture, and mononuclear cell infiltration compared with control group (Figure 1). However, there changes in APAP group were all reduced by tempol treatment, which may due to the protective effects of tempol on the hepatic cellular structures and membranes.

### Tempol Treatment Inhibits Oxidative Stress in Acetaminophen-Induced Acute Hepatotoxicity

**Figure 2:**
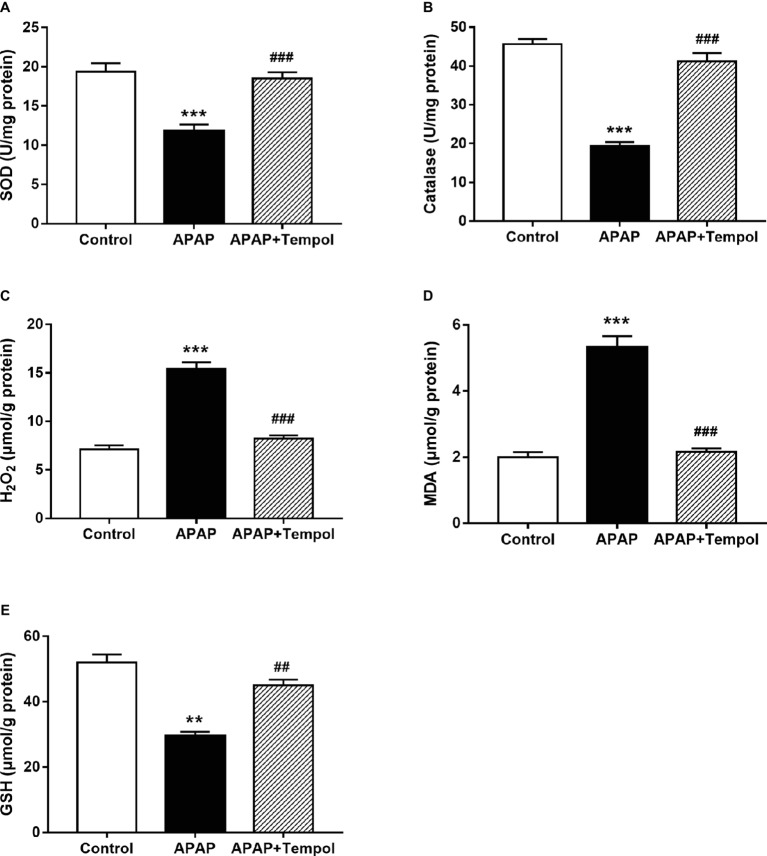
Tempol treatment inhibits oxidative stress in acetaminophen-induced acute hepatotoxicity. **(A)** Levels of SOD in liver from the control mice (control), vehicle-treated hepatotoxicity mice (APAP), and tempol-treated hepatotoxicity mice (APAP + tempol). **(B)** Levels of catalase in liver from three groups. **(C)** Levels of H_2_O_2_ in liver from three groups. **(D)** Levels of MDA in liver from three groups. **(E)** Levels of GSH in liver from three groups. Data are mean ± SEM. **p < 0.01 vs. control, ^##^*p* < 0.01 vs. APAP, ***p < 0.001 vs. control, ^###^*p* < 0.001 vs. APAP, *n* = 6.

Since acute liver injury is associated with excessive superoxide production, we tested levels of oxidative stress in liver tissues. Levels of SOD, catalase, and GSH were both reduced, while MDA and H_2_O_2_ were increased after treatment with APAP compared with control mice. However, all these changes were reversed by tempol treatment (Figures 2A–C, *p* < 0.001), which suggesting the inhibition of tempol on APAP-induced acute liver oxidative stress injury (Figure 2, *p* < 0.01).

### Tempol Protects Against Acetaminophen-Induced Acute Hepatotoxicity *via* the PI3K/Akt/Nrf 2 Pathway in Mice

**Figure 3:**
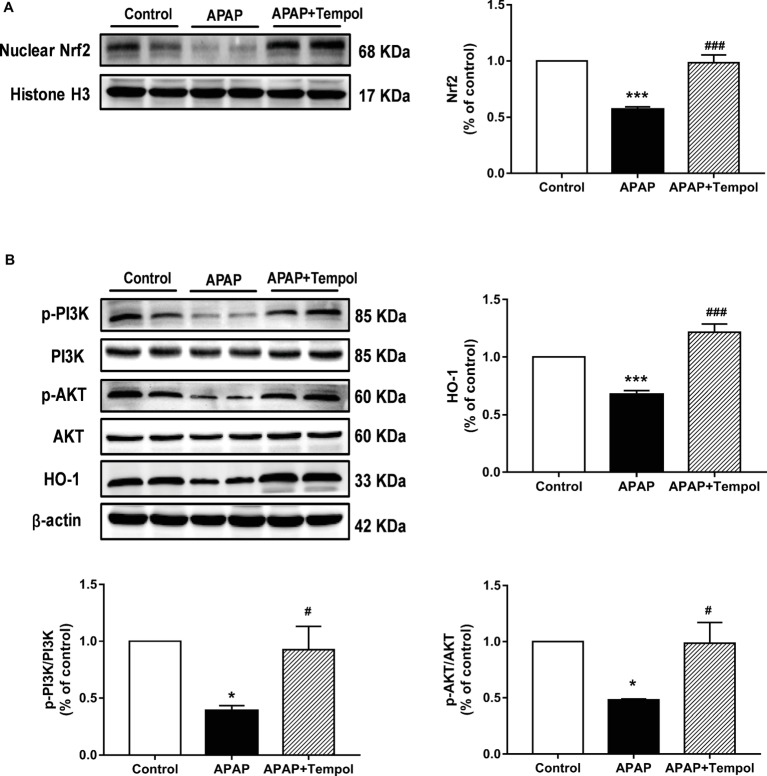
Tempol protects against acetaminophen-induced acute hepatotoxicity *via* the PI3K/Akt/Nrf2 pathway in mice. **(A)** Protein expression of Nrf 2 in the nucleus of liver from the control mice (control), vehicle-treated hepatotoxicity mice (APAP), and tempol-treated hepatotoxicity mice (APAP + tempol). **(B)** Protein expressions of PI3K, Akt, and HO-1 and phosphorylation of PI3K and Akt in liver from three groups. Data are mean ± SEM. *p < 0.05 vs. control, ^#^*p* < 0.05 vs. APAP, ***p < 0.001 vs. control, ^###^*p* < 0.001 vs. APAP, *n* = 4.

Compared with the vehicle-treated hepatotoxicity group, protein expression of Nrf 2 was 1.7-fold higher in liver nucleus from tempol-treated group (Figure 3A, *p* < 0.001). This matched with the increased antioxidant proteins in liver from tempol-treated group (Figure 2). To further explore the underlying mechanism, we evaluated phosphorylation of PI3K and Akt in hepatic tissues. Western blots showed that phosphorylation expressions of PI3K and Akt were decreased in vehicle-treated liver injury group, whereas tempol treatment upregulated both (Figure 3B, *p* < 0.05). Furthermore, results showed that protein expression of HO-1 was increased in tempol-treated group (Figure 3B, *p* < 0.001). These indicated that tempol protects against acetaminophen-induced acute hepatotoxicity *via* the PI3K/Akt/Nrf2/HO-1 pathway in mice.

### Tempol Treatment Decreases Hepatic Apoptosis in Acetaminophen-Induced Acute Hepatotoxicity

**Figure 4:**
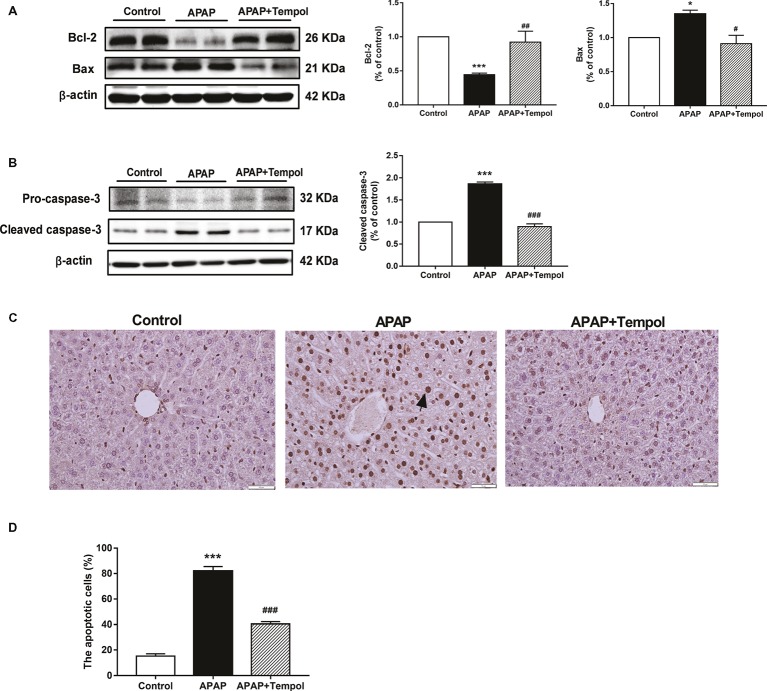
Tempol treatment decreases hepatic apoptosis in acetaminophen-induced acute hepatotoxicity. **(A)** Protein expressions of Bcl-2 and Bax in liver from the control mice (control), vehicle-treated hepatotoxicity mice (APAP), and tempol-treated hepatotoxicity mice (APAP + tempol). **(B)** Protein expressions of pro-caspase-3 and cleaved caspase-3 in liver from three groups. **(C)** Tunel-staining of hepatic tissues from three groups. The black arrow indicates typical apoptotic cells. Scale bar = 50 μm. **(D)** Percentage of TUNEL-positive cells evaluated by image analyzer. Data are mean ± SEM. *p < 0.05 vs. control, ^#^*p* < 0.05 vs. APAP, ^##^*p* < 0.01 vs. APAP, ***p < 0.001 vs. control, ^###^*p* < 0.001 vs. APAP, *n* = 4.

We used TUNEL staining to evaluate apoptotic cells in liver. The nuclei were lightly counterstained with hematoxylin. Apoptotic cells with nuclei staining brown were recognized as TUNEL-positive cells excluding necrosis. Numbers of TUNEL-positive tubular cells were quantified by counting 10 randomly chosen, non-overlapping fields per slide. Results showed that TUNEL-positive cells were markedly increased in APAP group compared with control group. However, tempol treatment reversed this change (Figure 4C, *p* < 0.001), which suggested that tempol could protect against APAP-induce hepatic apoptosis. To understand how activation of tempol contributes to hepatic apoptosis, we tested protein expression of apoptotic proteins. Compared with the vehicle-treated group, tempol administration decreased pro-apoptotic proteins (cleaved caspase-3 and Bax) in the liver (Figure 4B, *p* < 0.01). Also, expression of Bcl-2, as an anti-apoptotic protein, was increased in hepatic tissue from tempol-treated group. (Figure 4A, *p* < 0.01). These further supported that tempol had a vital role in inhibiting apoptosis in APAP-induced liver injury.

### The Proposed Mechanism for the Protective Effect of Tempol Against Acetaminophen-Induced Acute Hepatotoxicity in Mice

**Figure 5:**
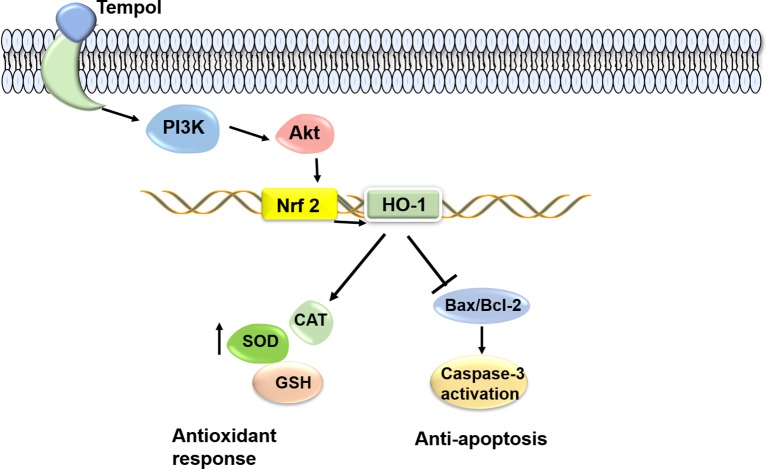
The proposed mechanism for the protective effect of tempol against acetaminophen-induced acute hepatotoxicity in mice. Tempol administration in APAP-induced acute hepatotoxicity mice normalizes liver function *via* activating PI3K/Akt/Nrf2 pathway, thus increasing antioxidant proteins and inhibiting hepatic apoptosis.

Tempol administration in APAP-induced acute hepatotoxicity mice normalizes liver function *via* activating PI3K/Akt/Nrf2/HO-1 pathway, thus increasing antioxidant proteins and inhibiting hepatic apoptosis (Figure 5).

## Discussion

The present study was aimed to discuss the effects of tempol on APAP induced acute hepatotoxicity. Our results showed that tempol treatment significantly improved serum levels of liver enzymes and liver histology changes induced by APAP. Tempol treatment upregulated antioxidant proteins, including SOD, catalase, and GSH, while reduced H_2_O_2_ and MDA in liver. Compared with the vehicle-treated hepatotoxicity group, protein expression of Nrf 2 and HO-1 and phosphorylation of PI3K and Akt were all increased in liver from tempol-treated group. In addition, western blots showed that tempol administration decreased pro-apoptotic protein expressions (cleaved caspase-3 and Bax) and increased anti-apoptotic Bcl-2 in liver, as well as reducing apoptotic cells of TUNEL staining, which suggested apoptotic effects of tempol treatment. Overall, our results show that tempol treatment improved APAP-induced acute hepatotoxicity by activating PI3K/Akt/Nrf2 pathway, thus enhancing antioxidant response and inhibiting hepatic apoptosis in mice.

In light of previous studies, mitochondrial oxidative stress is regarded as the dominant cellular event in APAP-induced acute hepatotoxicity (Wang et al., 2016a; Yan et al., 2018). Several natural compounds have been reported to protect against APAP-induced acute liver injury *via* antioxidant capacity (Rasool et al., 2010; Ding et al., 2016; Rashid et al., 2016). In our study, tempol treatment upregulated levels of antioxidant proteins, including SOD, catalase and GSH, thus inhibiting oxidative stress in APAP-induced liver injury. Although tempol can directly metabolized superoxide and hydrogen peroxide, tempol has also been reported to reset the anti-oxidant defense system through other pathways (Wilcox and Pearlman, 2008). Interestingly, our study found that tempol treatment upregulated Nrf 2 expression and its downstream gene HO-1, which may contribute to its antioxidant effect. Since the PI3K/Akt signaling pathway has been reported to be related with Nrf-2-dependent transcription (Balogun et al., 2003) and HO-1 expression (Shen et al., 2004). In the present study, we found that phosphorylation of PI3K and Akt were both increased by tempol. These indicated that tempol protected against APAP-induced hepatotoxicity *via* activating the PI3K/Akt/Nrf2/HO-1 pathway. Similarly, previous reports have shown that tanshinone IIA and caffeic acid protected against acetaminophen-induced hepatotoxicity through activating the Nrf 2 signaling pathway (Pang et al., 2016; Wang et al., 2016b), which indicated activation of Nrf 2 may be regarded as a potential target for the treatment of acute liver injury.

Previous research had indicated that APAP-induced hepatocyte apoptosis also plays an important role in the pathogenesis of acute liver injury (Jaeschke et al., 2012; McGill et al., 2012). Apoptosis is concerned as a process of programmed cell death. Bcl-2 family regulates mitochondria-dependent apoptosis, including both anti-apoptotic proteins (Bcl-2) and pro-apoptotic protein (Bax) (Martinou and Youle, 2011). Both of them regulate the downstream caspase-3, which is a center executioner caspase provoking proteins cleavage and leading cell apoptosis (Kitazumi and Tsukahara, 2011). Our study showed that expression of Bcl-2 was reduced and cleaved caspase-3 and Bax were both upregulated after APAP injection. Interestingly, these changes were markedly ameliorated by tempol, as well as reducing an increase of TUNEL positive staining, labeling of apoptotic cells. These suggested the antiapoptotic effect of tempol. Previous studies have indicated that oxidative stress was regarded as a trigger for activating apoptosis signaling pathway (Piao et al., 2012; Chen et al., 2014). Therefore, inhibition of apoptosis by tempol treatment, may partly due to the activation of Nrf-2 related pathway and the prevention of oxidative stress by tempol. In addition, PI3K/Akt pathway was concerned to regulate apoptosis and survival (Fry, 1994; Kandel and Hay, 1999) and recently has been implicated in the protection of liver damage (Sano et al., 2010). Therefore, activation of PI3K/Akt signal by tempol treatment also contributes to the anti-apoptosis effects.

In conclusion, we are the first to evaluate the effect of tempol on APAP-induced acute hepatotoxicity in mice. A vital finding is that we found tempol treatment in APAP-induced acute hepatotoxicity mice normalizes liver function mainly *via* activating PI3K/Akt/Nrf2/HO-1 pathway, which results in anti-oxidative stress and anti-apoptotic effects, suggesting a potential treatment with tempol to APAP-induced acute liver injury.

## Data Availability

The raw data supporting the conclusions of this manuscript will be made available by the authors, without undue reservation, to any qualified researcher.

## Author Contributions

All authors have seen and approved the final version of the manuscript. JH and ZG conceived and designed the studies. ZG and CW performed most of the experiments with animal tissues, the statistical analysis, and contributed intellectually to the writing of the manuscript. XL made the animal model. JZ contributed to the conception of the article, the data interpretation, drafting of the manuscript, and revised the article for important intellectual content.

### Conflict of Interest Statement

The authors declare that the research was conducted in the absence of any commercial or financial relationships that could be construed as a potential conflict of interest.
